# Report of the Institution for the Cure and Prevention of Contagious Fever in the Metropolis, 5th of May, 1805

**Published:** 1805-10-01

**Authors:** 


### Report of the Institution for the Cure and Prevention of Contagious Fever in the Metropolis, 5th of May, 1805.

| | |
|---|---|
| Admitted May 1804, to May 1805, | 80 |
| Dismissed cured - - - - - - - | 71 |
| Dismissed as an improper object - | 1 |
| Died - - - - - - - - - - - - | 7 |
| Died (not of Fever) - - - - - | 1 |

During the last year, and especially in the autumnal months, the metropolis has been, in an extraordinary degree, free from contagious fever: hence the number of patients, admitted into the House of Recovery since the date of the last Report, is scarcely equal to one half of that received during the year preceding. This diminution of the common evil of the crouded and uncleanly parts of the town, is perhaps too general, to be attributed solely to the efforts which have been made by the Fever Institution. It is connected probably with a comfortable supply of provisions and of fuel among the poor, and and consequently with the means of observing a greater degree of cleanliness, and with fewer inducements to shut out the cold air from their apartments: and probably it may also depend to a certain degree, on some unassignable state of the atmosphere: yet, upon the whole, the beneficial operations of the Institution have been extremely manifest. The district with which the house belonging to the Institution is, by situation, more immediately connected, has been, and still continues more free from typhous fever, than the more distant parts of the metropolis. Several of the courts within this district, especially Spread Eagle Court, Gray's Inn Lane, some of the courts and alleys in the vicinity of Field Lane, Saffron Hill, &c. at first furnished a large proportion of the patients, who were admitted to the benefits of the Institution. These courts have been gradually purified under its direction by whitewashing, fumigation, and the removal of the infected persons; so that during the last year, only twenty patients have been found in this district, and three fourths of those admitted were brought from distant parishes, chiefly from Cripplegate, and the neighbourhood of Spitalfields, and partly from the Borough. The efficacy of these means of purification, and the facility with which contagion is destroyed, and the progress of malignant fevers arrested, is thus illustrated in a striking manner. Where the ordinary aid of public charities is resorted to by patients in fever, it has been observed with regret, that they frequently suffer a return of the disease, in consequence of the operation of the contagion, which the first attack had produced. But in those houses in which the plan of purification, adopted by the Fever Institution, has been employed, no relapses of this kind have occurred, nor has the disease been known thenceforward to extend itself to any individuals not previously infected. The houses which were purified during the year, comprised in the last Annual Report, have not afforded any instances of the disease during the present year; and even some of the courts above referred to, which were almost generally cleansed in the former period, have produced no patients for the House of Recovery in the latter period. In the course of the year ending this day, *thirty-six* apartments have been whitewashed and fumigated, and fumigation has been employed in almost all the apartments from which patients have been received. It is to be lamented that, in a few instances, the offer of whitewashing has been refused.

 It

It may be farther remarked, that, while on the one hand the practices of the Institution have demonstrated the readiness with which contagion may be suppressed or destroyed, they have on the other afforded no less satisfactory evidence of the facility of confining its operations within very narrow limits. The House of Recovery, occupied for the purposes of the Institution, stands in the midst of a row, in contact with dwelling houses on both sides; four hundred and twenty patients have been received into it; there has been an almost uninterrupted succession of fevers within its chambers; yet the neighbourhood has continued altogether free from the disease; no complaint has been communicated from it in any way; and the apprehensions of the adjoining inhabitants, which common prejudices with respect to the nature of contagion, at first naturally excited, seem to have now altogether subsided. This is an additional fact of considerable importance, when conjoined with the experience of Dr. Ferriar at Manchester, and tends to prove that contagion is not conveyed widely through the air. Were not the general prejudice on this subject strong, this fact indeed might have been clearly anticipated. For if, as we have learnt from experience, contagious *effluvia*, diluted by a free admission of air, are not communicated from room to room in a house, nor even from bed to bed in the wards of an hospital, it scarcely required a positive experiment to prove that houses, even in contact, were not liable to infect each other.

The mortality, during the last year, has been somewhat less in proportion than in the year preceding: but the deaths of the year 1803, were a little over rated by our late able and benevolent physician, Dr. Dimsdale, from a desire of observing an extreme candour in his report, which led him to include in the list two or three instances of fatality produced by diseases unconnected with fever. On the other hand, it should not be omitted that a few of the cases received into the House in the course of the last autumn, were of such a mild nature, as to render it doubtful whether they were merely the consequence of cold, and did not generate contagion, or were strictly of a typhous nature. All the patients who died, with one exception, were admitted on or after the seventh day; two so late as the twelfth and thirteenth days. The case excepted was one of those unfortunate combinations of disease, which almost elude the powers of medicine; namely, a typhoid fever, conjoined with an extensive inflammation mation of the lungs; the patient was admitted on the third, and died on the fifth day of the disorder. The body was examined, and the previous idea of the disease was confirmed by the dissection. The patient who died some time after her recovery from fever, was cut off by the return of a complaint, to which she had formerly been subject, a *tympanites*, which her reduced strength was unable to resist. The child, dismissed as an improper object of the Institution, laboured under a severe hooping cough; she was received in consequence of a recommendation from an apothecary, stating that her complaint was fever.

From the nature of this Report, it cannot be considered as a proper medium for the communication of medical facts or observations; but since the subject of cold bathing, as a remedy in fever, has been alluded to in former Reports,* it may not be improper to add, that the practice has been resorted to during the last year, in every case, in which circumstances required or admitted it. Patients are scarcely ever received into the House before the fourth day of the fever, and therefore the chance of experiencing the full effects of the practice, viz. of arresting at once the progress of the disease, is not very great. But it has still appeared to be a valuable expedient, in alleviating some of the most distresing symptoms of fever; such as the pungent heat, thirst, delirium, and watchfulness, which, though the effects of the febrile state in the first instance, become secondarily the cause of its prolongation and danger, by the irritation which they produce. It has generally been found to be grateful to the sensations of the patient, and has been commonly followed by a refreshing sleep, and a cooling perspiration; and it has not, in one instance, been attended with any unpleasant consequences.

STATEMENT OF THE ACCOUNTS.

| RECEIPTS. | £. | s. | d. |
|---|---|---|---|
| Balance from last year's account - - - - - - | 278 | 15 | 11 |
| Donation from the Sub-Committee, (appointed by the Committee of the Society for bettering the Condition of the Poor) for Prevention of contagious Fevers - - - - - - - - - - - - | 300 | 0 | 0 |
| Carried over - £. | 578 | 15 | 11 |

* See "Extract from an account of cases of Typhus Fever in which the affusion of cold water has been applied in the London House of Recovery."

| | £. | s. | d. |
|---|---|---|---|
| Brought over - | £.578 | 15 | 11 |
| Donations from the 5th of May, 1804, to the 2d of May, 1805 | 15 | 10 | 0 |
| Annual Subscriptions, &c. and Dividends of £.3 per Cent. Consols, received within the same time | 603 | 6 | 0 |
| | 1197 | 11 | 11 |

| DISBURSEMENTS. | £. | s. | d. |
|---|---|---|---|
| Rent, Taxes, and Insurance | 54 | 0 | 9 |
| Housekeeping, Matrons', Inspectors', Nurses', and Servants' Wages, also Furniture, Repairs, and clothing for Patients | 241 | 14 | 4 |
| Drugs | 19 | 14 | 10 |
| Removing Patients from their Habitations, fumigating, whitewashing, and cleaning infected apartments | 23 | 14 | 2 |
| Gratuities and Salaries | 172 | 10 | 0 |
| Printing, Advertising, Stationary, and Stamps | 38 | 0 | 5 |
| Paid for Room to meet in, and £. 4. 4*s*. for the delivery of Dr. Stanger's Pamphlets to the Members of Parliament | 4 | 11 | 6 |
| Invested in the Purchase of £. 3 per Cent. Consols. in the Names of the Trustees, and Broker's Commission | 291 | 5 | 0 |
| Sundries | 0 | 2 | 10 |
| Balance in the Treasurer's hands | 351 | 18 | 1 |
| | 1197 | 11 | 11 |

